# A Novel Addressing Scheme for PMIPv6 Based Global IP-WSNs

**DOI:** 10.3390/s110908430

**Published:** 2011-08-29

**Authors:** Md. Motaharul Islam, Eui-Nam Huh

**Affiliations:** Department of Computer Engineering, College of Electronics and Information, Kyung Hee University, Yongin-si 446-701, Korea; E-Mail: motahar@khu.ac.kr

**Keywords:** PMIPv6, IP-WSN, IETF, 6LoWPAN, IEEE 802.15.4, IP-sensor node

## Abstract

IP based Wireless Sensor Networks (IP-WSNs) are being used in healthcare, home automation, industrial control and agricultural monitoring. In most of these applications global addressing of individual IP-WSN nodes and layer-three routing for mobility enabled IP-WSN with special attention to reliability, energy efficiency and end to end delay minimization are a few of the major issues to be addressed. Most of the routing protocols in WSN are based on layer-two approaches. For reliability and end to end communication enhancement the necessity of layer-three routing for IP-WSNs is generating significant attention among the research community, but due to the hurdle of maintaining routing state and other communication overhead, it was not possible to introduce a layer-three routing protocol for IP-WSNs. To address this issue we propose in this paper a global addressing scheme and layer-three based hierarchical routing protocol. The proposed addressing and routing approach focuses on all the above mentioned issues. Simulation results show that the proposed addressing and routing approach significantly enhances the reliability, energy efficiency and end to end delay minimization. We also present architecture, message formats and different routing scenarios in this paper.

## 1. Introduction

Recent technological advances in wireless communication and electronics have developed sensor nodes containing low cost, low power, multifunctional sensor nodes that are small in size and can communicate over short distances [1]. In the past, applications of sensor networks were thought to be very specific. The communication protocols of sensor network were also very simple and straightforward. The research communities were even against the use of the internetworking protocols in WSNs. There are reasons behind this, such as the resource constraints for layered architecture, the problems of configuring large numbers of devices, the essence of sensor nodes’ distinct identity, *etc.*, but with the advent of the Internet of things and federated IP-WSNs, this concept is going to become blurred. The huge number of IPv6 addresses, the necessity for end to end communication and the advances in micro-electronics have changed the concepts of the research community [2]. Nowadays a tiny sensor node can hold a compatible TCP/IP protocol stack [3], so we can now think of using the concept of internetworking protocols in IP-WSNs [4].

IP-WSN nodes are expected to vastly outnumber conventional computer hosts, so we can easily think of providing IPv6 addresses to individual sensor nodes since it provides around 6 × 10^23^ addresses per square meter of the Earth’s space. The IPv6 over low power wireless personal area network (6LoWPAN) working group of the Internet Engineering Task Force (IETF) defines the manner in which IPv6 communication is to be carried out over IEEE 802.15.4 interface [5,6]. Although 6LoWPAN helps in making the wide implementation of IP-WSN a reality and its end to end communication to the external world feasible, the excessive signaling costs for sensor nodes because of too much tunneling through the air makes it difficult. Excessive signaling costs therefore become a barrier for the application of IP-WSNs, especially in the case of the mobility scenario of individual sensor nodes or groups of nodes in different areas such as in a patient’s body sensor network, in industrial automation, *etc*. [5]. Nowadays most communication protocols are host based, which is practically infeasible for IP-WSNs since individual nodes need to participate in mobility related signaling. On the other hand, addressing of individual sensor nodes in IP-WSNs is necessary for the end to end communication. The tiny sensors are not capable of holding the complete IPv6 addressing since the IEEE 802.15.4 packet size is 127 octets [6,7] and it is not feasible to contain the routing table for the individual IP-WSN sensor node. These are the major problems to be addressed in IP-WSNs.

With this in mind, in our previous work [8] we focused on the issue of mobility in IP-WSNs with the intention of developing an energy efficient network-based communication protocol for IP-WSNs. We proposed the Sensor Proxy Mobile IPv6 (SPMIPv6), which is a network-based protocol that provides mobility support to any IPv6 host within a restricted and topologically localized portion of the network, but in our previous paper we did not focus in detail on the addressing and routing issues. In this paper we propose a hierarchical addressing and layer three routing protocol for IP-WSNs. In the proposed addressing and routing protocol individual IP-WSN nodes will be identified by a unique global IPv6 address. This global IPv6 address will be generated in a hierarchical approach.

IP-WSN nodes known as Reduced Function Devices (RFDs) will be identified within a particular Wireless Personal Area Network (WPAN) domain by a 16 bit short address. The Sensor Mobile Access Gateway (SMAG) known as a Full Functional Device (FFD) will hold IEEE EUI-64, and finally a Border Router (BR) will hold 128 bits global unicast addressing. The RFD is assigned a 16 bit short address, which is unique within a WPAN or SMAG domain, and remains fixed irrespective of its location within the WPAN. All three levels of addresses are created hierarchically.16 bits short addresses are assigned to a RFD at the time of deployment. A EUI-64 identifier is formed based on the 16 bits short address, and finally 128 bits global addressing is based on the EUI-64 bits identifier. This addressing scheme will uniquely identify the IP-WSN nodes and at the same time reduce different types of consumption. The main contributions of this paper can be summarized as follows:

- A global IP-WSN architecture is proposed, by which we introduce our addressing and routing scheme.
- We propose a global addressing scheme for the individual IP-WSN nodes which is generated in a hierarchical fashion. We also propose a routing scheme based on the hierarchical addressing.
- We propose software architecture, and the respective message formats, network model and evaluate the performance of the proposed protocol architecture.
- We address the applicability of mobility issues of individual or a group of IP-sensor nodes in a global patient care scenario. We propose the addressing format for the patient care scenario considering different mobility perspectives. We also focus on the routing scenario in all the mobility cases.
- Mathematical analysis and simulation experiments are conducted to show the effectiveness of the proposed scheme. Our simulation results show that the proposed scheme effectively reduces the signaling and mobility cost, end to end delay in terms of the number of IP-WSNs and hops as compared to MIPv6 and PMIPv6, respectively. There is also a significant improvement in energy consumption for data payload and IP-WSN node density in the network.

The rest of the paper is organized as follows: Section 2 reviews the background related to PMIPv6 and 6LoWPAN. The proposed protocol architecture, along with IP-WSN based patient care application, mobility scenarios, addressing details and message formats are presented in Section 3. Section 4 describes performance analysis and evaluation, Section 5 shows the experimental and simulation results, and finally Section 6 concludes the paper.

## 2. Background

### 2.1. Overview of PMIPv6

The foundation of PMIPv6 is based on MIPv6 in the sense that it extends MIPv6 signaling and reuses many concepts such as the home agent (HA) functionality. However, PMIPv6 is designed to provide network-based mobility management support to a Mobile Node (MN) in a topologically localized domain. Therefore, an MN is free from participation in any mobility-related signaling, and the proxy mobility agent in the serving network performs mobility-related signaling on behalf of the MN. Once an MN enters its PMIPv6 domain and performs access authentication, the serving network ensures that the MN is always on its home network and can obtain its home address on any access network. The serving network assigns a unique home network prefix to each MN, and conceptually this prefix always follows the MN wherever it moves within a PMIPv6 domain. From the perspective of the MN, the entire PMIPv6 domain appears as its home network. Accordingly, it is needless to configure the care of address at the MN. The new functional entities of PMIPv6 are the mobile access gateway (MAG) and local mobility anchor (LMA). The MAG typically runs on the access router (AR). The main role of the MAG is to detect the MN’s movements and initiate mobility-related signaling with the LMA on behalf of the MN. In addition, the MAG establishes a tunnel with the LMA for enabling the MN to use an address from it home network prefix and emulates the MN’s home network on the access network for each MN. On the other hand, the LMA is similar to the HA in MIPv6. However, it has additional capabilities required to support PMIPv6. The main role of the LMA is to maintain reachability to the MN’s address while it moves around within a PMIPv6 domain, and the LMA includes a binding cache entry for each currently registered MN. The binding cache entry maintained at the LMA is more extended than that of the HA in MIPv6 with some additional fields such as the MN identifier, the MN’s home network prefix, a flag indicating a proxy registration, and the interface identifier of the bidirectional tunnel between the LMA and MAG. Such information associates an MN with its serving MAG, and enables the relationship between the MAG and LMA to be maintained [9].

### 2.2. 6LoWPAN

The 6LoWPAN [10] working group of the IETF has defined an adaptation layer for sending IPv6 packets over IEEE 802.15.4. The goal of 6LoWPAN is to reduce the sizes of IPv6 packets to make them fit in 127 byte IEEE 802.15.4 frames. The 6LoWPAN proposal consists of a header compression scheme, a fragmentation scheme, and a method for framing IPv6 link local addresses into IEEE 802.15.4 networks. The proposal also specifies enhanced scalabilities and mobility of sensor networks. The challenge to 6LoWPAN lies in the sizable differences between an IPv6 network and an IEEE 802.15.4 network. The IPv6 network defines a maximum transmission unit as 1,280 bytes, whereas the IEEE 802.15.4 frame size is 127 octets. Therefore, the adaptation layer between the IP layer and the MAC layer must transport IPv6 packets over IEEE 802.15.4 links. The adaptation layer is responsible for fragmentation, reassembly, header compression and decompression, mesh routing, and addressing for packet delivery under the mesh topology. The 6LoWPAN protocol supports a scheme to compress the IPv6 header from 40 bytes to 2 bytes [11].

## 3. Proposed Protocol Architecture

### 3.1. Protocol Architecture

**Figure 1.**
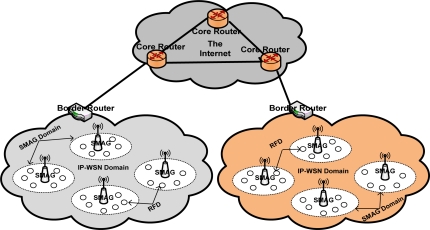
Global IP-WSN Architecture.

To introduce efficient addressing and routing scheme in our proposed global IP-WSN, we use sensor proxy mobile IPv6 (SPMIPv6) architecture [8]. The proposed architecture has different functional components. The architecture mainly consists of a sensor network-based localized mobility anchor (SLMA). Here we use SLMA as a border router (BR) since it provides an interface between the IP-WSN domain and the traditional Internet domain. It also contains a sensor network-based mobile access gateway (SMAG). For the end-to-end communications, it contains many full functional devices (FFDs) and reduced functional devices (RFDs). This architecture is designed in a hierarchical approach. We discuss the individual components of the proposed architecture in more detail in the following subsections with the help of Figure 1. Figure 1 consists of two IP-WSN domains which are connected to the Internet domain by a BR. The IP-WSN domains again consist of different SMAG domains. The SMAG domains further consists of FFDs and RFDs.

#### 3.1.1. Border Router

The border router acts as a topological anchor point for the entire IP-WSN domain. The main role of the border router is to maintain accessibility to the sensor node while the node moves within or outside the IP-WSN domain. The border router includes a binding cache entry for each sensor node, encapsulation and decapsulation sections and a SMAG information table. The binding cache entry at the border router is used for holding the information of the mobile sensor node. It includes different information such as a sensor node’s address, the sensor node’s home network prefix, and a flag bit indicating sensor proxy registration.

The border router has sufficient memory to hold all of these necessary records. It has sufficient power supply and processing capability. It also acts as the interfacing device between IP-WSN domain and the Internet domain. As an interfacing entity it facilitates the communication of IP-WSN with the Internet world. In this scheme, authentication, authorization, and accounting (AAA) service have been integrated within border router. We name this integrated service a Sensor network-based Aauthentication, Authorization, and Accounting (SAAA) scheme. The SAAA scheme helps the SMAG and sensor nodes achieve secured mobility in the IP-WSN domain. It also facilitates authentication services for each fully functional sensor node.

#### 3.1.2. SMAG

The SMAG acts like a sink node in a traditional sensor network. In the proposed IP-WSN based on SPMIPv6 it acts like an access gateway router with the main function of detecting sensor node movement and initiating mobility-related signaling with the sensor node’s border router on behalf of the sensor node. It can move with its member sensor node as a SMAG domain within or outside a IP-WSN domain similar to the body sensor network of a patient. It consists of different functional modules such as routing, neighbor discovery, sensor information table, adaptation module and interfacing modules to the sensor node and border router. The routing module performs the efficient data transmission among individual sensor nodes and facilitates the end to end communication. The neighbor discovery module performs neighbor discovery and duplicate address detection functionality. The adaptation module performs the task of transmitting IPv6 packets over IEEE 802.15.4 link as mentioned in the 6LoWPAN adaptation layer. The sensor information table provides the up to date information about the sensor nodes to the border router. It works with the close association of the binding cache entry of the border router, and the two interfacing modules communicate with the border router and the sensor nodes. SMAG domain consists of FFD and RFD nodes. We also consider the SMAG domain as a personal area network (PAN) when it is regarded as a patient’s body area network in our global IP-WSN-based patient care unit described in Section 3.3.

#### 3.1.3. IP-Sensor Node

The IP-WSN domain consists of numerous sensor nodes based on IPv6 addresses. We consider the domain as a federated IP sensor domain. There are two types of sensor node. One type contains the tiny TCP/IP communication protocol stack with adaptation layer and IEEE 802.15.4 interface. This type can forward information to other node of similar type as well as information sensing from the environment. Actually this type of sensor node acts as a mini sensor router and is considered a fully functional device. The other type of sensor node has the protocol stack and environment sensing capability, but can only forward the sensed information to nearby mini sensor router nodes. These types of sensor nodes are considered reduced functional devices. Nevertheless, both types are able to perform end to end communication.

### 3.2. Software Architecture

**Figure 2.**
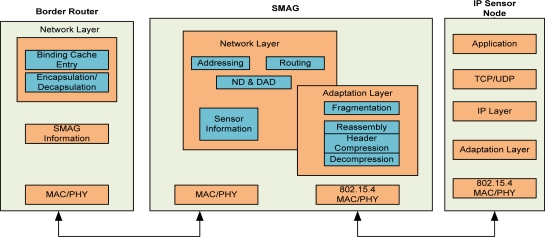
Software Architecture of global IP-WSN.

Figure 2 shows the detailed software architecture of the global IP-WSN which includes a border router, SMAG and IP-sensor nodes. It shows how these entities communicate each other by different types of interfaces. The SMAG needs two or more interfaces for the communication with different access networks such as IP-WSN domain and the Internet domain. It includes the network layer, adaptation layer and physical layer functionality. The network layer provides different functionality such as addressing, routing, neighbor discovery and the data structure for holding IP sensor node information. The most important layer is the adaptation one that ensures mesh routing, compression & decompression, and fragmentation and reassembly.

The physical layer provides access to different physical interfaces. The border router holds network related information such as binding cache entry, encapsulation and decapsulation. The binding cache entry provides the data structure to hold different information such as new IP-WSN flags, addresses for each interface, home prefixes, bi-directional tunnel interface identifiers, access technology and time stamps. Finally the IP sensor nodes work as a very smart tiny 6LoWPAN based node. They hold tiny TCP/IP protocol stacks. Moreover they hold the basic functionality of the adaptation layer. All the sensor nodes consist of IPv6 addresses for local and global communications.

### 3.3. IP-WSN Based Patient Care Scenario

**Figure 3.**
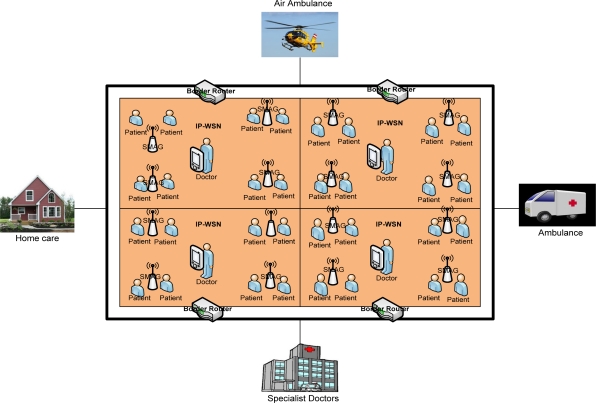
Global IP-WSN based patient care unit.

IP-WSN is envisioned to be heavily used in healthcare environments. Although hospital scenarios can be handled in different ways, IP-WSN provides great potential to facilitate the development of new services by getting rid of cumbersome wires and simplifying patient care in hospitals and for home care as the World is rapidly graying. The speed with which this age-structural change is taking place implies an urgent need for solutions that will relieve the mounting pressure on our health-care systems as well as support a better quality of life and quality of care for our aged. With this in mind, we depict an IP-WSN based patient care unit which is shown in Figure 3. We consider here a multipurpose specialized hospital. On different floors of the hospital there are different types of patient care units. All the floors are considered a single SPMIPv6 domain. Inside the SPMIPv6 domain there are different floors which consist of four separate IP-WSN domains. Each IP-WSN domain again contains different SMAG domains.

Each domain is under the custody of a border router. All the border routers of the different IP-WSN domains are connected by high speed connectivity. Sensor nodes are deployed on the body of the patients as well as all over the surrounding environment. Doctors are always monitoring the patients online. A patient can get special care both in the hospital and personal home care. In case of emergency a patient can also get medicare from running ambulance, from air ambulance and from a remote specialized hospital. The patient care unit ensures real time care and observation of the patients from a central specialized doctor’s forum. In an emergency, patients can move from one place to another with their complete set-up so that seamless connectivity with the sophisticated medical equipment remains established and doctors can monitor the patient online from the central doctor’s research group.

### 3.4. Mobility Scenario in IP-WSN

We consider a state-of-the-art technology based patient care unit in a specialized hospital to depict different types of mobility scenarios [12]. We describe the patient care unit of the hospital environment in Section 3.3 with the help of Figure 3. The mobility issues considered in this scenario are: Case-I: Movement of nodes within the same SMAG domain, Case-II: Movement of nodes between different SMAGs of the same IP-WSN domain, Case-III: Movement of nodes between different SMAGs of different IP-WSN domains, Case-IV: Movement of a SMAG domain within the same IP-WSN domain, Case-V: Movement of a SMAG domain between different IP-WSN domains, Case-VI: Patient monitoring in personal home environment. These scenarios are explained below:

Case-I: In this case, the mobility of the nodes will be handled by the respective SMAG, without the involvement of the border router in the process. This is the simplest mobility scenario that arises frequently in hospital management. For example, a patient can move within the same SMAG domain of the hospital for different purposes such as for exercise and fresh air.
Case-II: In this case, mobility will be handled by the appropriate SMAG with minimal interaction from the border router. The initial coordination will be performed by the border router alone; then the SMAG will oversee the remaining procedures. In our patient care scenario, a patient can move from one SMAG domain to another in the same IP-WSN domain of the patient care unit.
Case-III: In this case, mobility is among different IP-WSN domains, sometimes using the public Internet domain. In the proposed patient care unit, a patient can move on an emergency basis from one IP-WSN domain of a hospital to another IP-WSN domain of the same hospital.
Case-IV: In this case, the scenario is based on the network mobility (NEMO) protocol [13]. It is confined to the same IP-WSN domain. In our patient care scenario, a patient with the whole set up can move from one location to another location within the same IP-WSN domain. In this scenario a patient’s body is considered as a SMAG domain, so it is a SMAG domain movement within the IP-WSN.
Case-V: This case is also based on the NEMO approach. In this scenario a patient’s body is considered as a SMAG domain. In the patient care scenario, a patient with its setup considered as a SMAG domain can move from one IP-WSN to another IP-WSN, so it is SMAG domain movement among different IP-WSN domains.
Case-VI: Due to the increasing number of people in the aging demographic group, we consider this case so that a patient can be monitored continuously from the patient’s personal home care. In this case the home care unit will be fully automated with IP based sensors and other state of the art technology.

Although some of the mobility scenarios may seem complicated in the case of an IP-based wireless sensor network, still they have been considered an issue for future research. Because of the essence of huge deployment of IP sensor network in health care, departmental store and industrial automation, this mobility scenario will eventually come true in the near future.

### 3.5. Addressing Scheme

In our proposed addressing scheme, all the IP-WSN sensor nodes will be identified by the IPv6 global unicast address, but the 128 bits global unicast address will be generated in a hierarchical fashion. At the lowest level, individual RFD IP sensor nodes will be identified by 16 bits short addresses within a specific SMAG domain. These 16 bits short addresses will form the least significant 16 bits of the EUI-64 identifier. Then the SMAG/FFD IP sensor node will hold EUI-64 bits identifier. This EUI-64 bits identifier will be generated by a 48 bits prefix and a 16 bits short address. Finally for the global communication individual IP sensor node will be identified by a unique global unicast address which consists of a 64 bits prefix and a 64 bits EUI-64 identifier. In this scheme all the nodes can be uniquely identified from the outside world. This is a stateless addressing scheme. It also facilitates the routing mechanism since individual IP-WSN nodes need not contain any routing table information which consumes huge memory space and energy for the LoWPAN. Although EUI-64 bits identifier is considered to be globally unique, it is not possible to communicate end to end with the corresponding nodes and individual IP-WSN nodes. For this reason we need to deal with the global unicast address. In this scheme, FFD is regarded as SMAG and RFD is regarded as an end IP-WSN node.

#### 3.5.1. Intra-SMAG Communication

**Figure 4.**
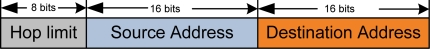
Addressing format to communicate within a SMAG.

An IP-WSN node will be uniquely identified by a 16 bits short address for local communication. This means communication within the SMAG domain. Here we assume that total number of nodes within an SMAG domain will not exceed 2^16^. Figure 4 depicts addressing format for inter-SMAG communication.

**Figure 5.**
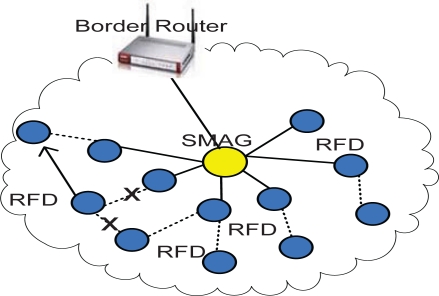
Intra-SMAG Communication.

This type of communication is required when transmission of information is carried out within RFD IP-sensor nodes. It just sends the message to the neighboring node. Finally it reaches the SMAG node. This reduces the energy consumption and communication overhead. In Figure 5, sensor nodes move from one link to another link within the same SMAG domain. This is identified by the re-association procedure with another node and finally handled by the corresponding SMAG.

#### 3.5.2. Inter-SMAG Communication under Same Border Router

**Figure 6.**
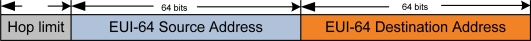
Addressing format to communicate among SMAGs under same border router.

Inter-SMAG communication will be performed by using EUI-64 bits addressing. In this case the EUI-64 bits identifier plays an important role in our addressing scheme by staying in between the 16 bits short address and 128 bits global unicast addressing. This addressing scheme will ensure inter-SMAG communication under the same border router. Figure 6 represents addressing format to communicate among different SMAGs under same border router.

**Figure 7.**
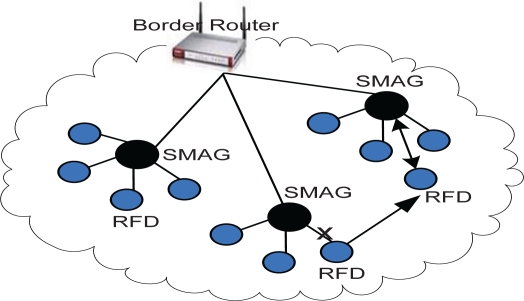
Inter-SMAG communication under same border router.

In Figure 7, a sensor is moving from one SMAG domain to another SMAG domain. This type mobility is identified by the new SMAG and the sensor node when the scanning process is performed. The new SMAG identify it by the duplicate address detection or from the pool of unassigned identifier. If the node is from the different SMAG domain then communication is performed among the peer SMAGs.

#### 3.5.3. Inter-SMAG Communication under Different Border Router

**Figure 8.**
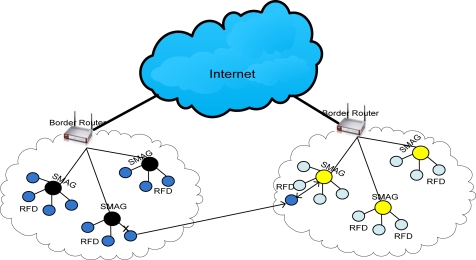
Inter-SMAG communication under different border router.

In this case the global unicast address will be used to communicate with the corresponding node. If the IP-WSN node moves from one SMAG domain under one border router to another SMAG domain under another border router then it will get a care of address for the time being for the immediate connectivity to the corresponding node. After the discovery of the home border router address new location of the IP-WSN will be reintroduced and further communication will be performed by the addressed formed by the EUI-64 address identifier and foreign router prefix. The scenario is depicted in Figure 8.

**Figure 9.**
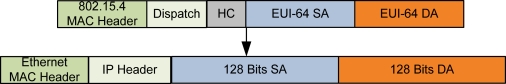
Communication from IP-WSN to CN.

**Figure 10.**
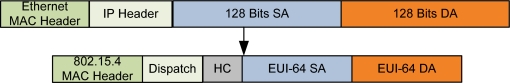
Communication from CN to IP-WSN.

In Figures 9 and 10, we demonstrate the addressing format to communicate from IP-WSN to CN (Figure 9) and CN to IP-WSN, as shown in the Figure 10.

### 3.6. Proxy Binding Message Format

**Figure 11.**
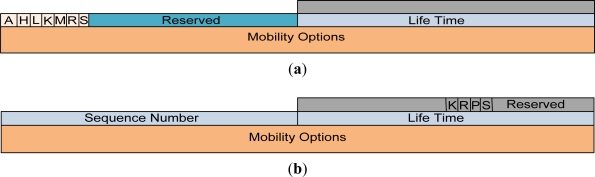
(**a**) PBU Message Format; (**b**) PBA Message Format.

In the proposed proxy binding update (PBU) and proxy binding acknowledgement (PBA) messages in Figure 11, we have specified a flag bit S. If this S flag is set, it indicates an IP-WSN based on SMIPv6 operations. If the S bit is not set, this indicates non-SPMIPv6 operations. The meanings and descriptions of the other flags are described in different requests for comments (RFCs) [9,13–15]. The mobility options header plays an important role for the different types of mobility scenario. Some of the mobility header options are hand-off indicator options, access technology type options, link layer identifier options, link local address options and time stamp options.

### 3.7. Border Router Solicitation and Advertisement Message Format

**Figure 12.**
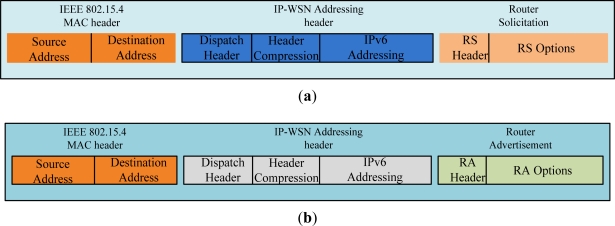
(**a**) IP-WSN Router Solicitation (RS) Message Format; (**b**) IP-WSN Router Advertisement (RA) Message Format.

Figure 12 depicts the router solicitation (RS) and router advertisement (RA) message format of IP-WSN. Figure 12(a) shows the RS message which is sent by an IP-WSN sensor node. The IEEE 802.15.4 MAC header’s source and destinations address set to the source and destination sensor node’s MAC address. Dispatch of IP-WSN addressing header indicates the compressed IPv6 header. Header compression section compresses different fields of IP-WSN addressing header. Router solicitation message’s RS options enable IP-WSN gateway to obtain the sensor node’s MAC address, link-local address and sensor node’s ID. The RA message format has two options such as home network prefix (HNP) and address options. The HNP options are used to emulate a IP-WSN sensor node’s home network.

## 4. Performance Analysis and Evaluation

For analyzing signaling costs, we use a two-dimensional (2D) random walk model [16–18] based on the properties of regular, absorbing Markov chains. Random walk mobility models are designed for dynamic location areas (LAs) and are suitable for modeling user movement when mobility is generally confined to a limited geographical area. Such scenarios include homes, vehicles, hospitals, and department stores.

### 4.1. Network Mobility Model

**Figure 13.**
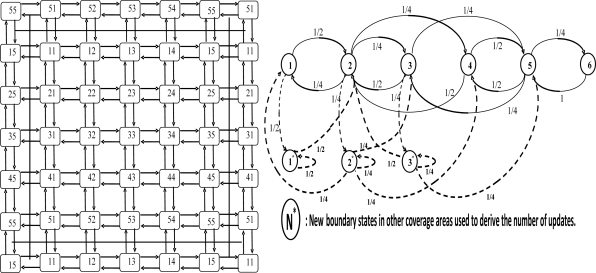
Square shaped cell layout of five-sublayer PAN area model & state diagram.

Mobility of IP-sensor node and the SMAG domain network are the major advantages of IP-WSN over conventional static wireless sensor network. In this paper, mobility is the key concern in the design and performance analysis of the IP-WSN. The mobility model plays a key role in studying different mobility management strategies such as registration, handoff, and authentication, *etc*. A mobility model with minimum assumptions and simple to analyze is very useful for IP-WSN. Most of wireless network performance studies assume that the coverage areas are configured as a hexagonal or square shape. We assume that IP-WSN networks are to be configured with hexagonal topology. Sensor nodes for an IP-WSN area are assumed to have identical movement patterns within and across IP-WSN. A 2D square shaped random walk mobility model can be used to study the movement pattern of the movable sensor nodes. In this paper, we will use a network model subject to some modification for the five-layer personal area network model, with n = 5. In our network model, an IP-WSN consists of a cluster of 2D square shape of sensor nodes, as shown in Figure 13 [15]. A brief description of Figure 13 is given in the following paragraph.

Figure 13 shows a square shaped micro-cell and macro-cell configuration. Each macro-cell covers n × n micro-cells (n = 5). Each macro-cell coverage area is one location area (LA). Firstly, we can aggregate the states of cells based on their symmetrical positions. Cells belonging to such aggregated state have the same properties. There are six aggregated states for the 5 × 5 square shaped micro-cell/macro-cells. The corner states (S_11_, S_15_, S_51_ and S_55_) are grouped into state S1. State S2 consists of (S_12_, S_14_, S_21_, S_25_, S_41_, S_45_, S_52_ and S_54_), state S3 consists of (S_13_, S_31_, S_35_ and S_53_), state S4 consists of (S_22_, S_24_, S_42_ and S_44_), state S5 consists of (S_23_, S_32_, S_34_ and S_43_), and state 6 consists of S_33_. In this figure, aggregate states S1, S2 and S3 are the boundary states.

We define asterisk boundary states, S1*, S2* and S3*, which are in the LAs adjacent to the LA under consideration. Figure 13 also demonstrates the state transition diagram for the Markov chain. Movement into any boundary state indicates inter-IP-WSN mobility, which can be used to study binding update costs.

### 4.2. Analysis of Signaling Costs

The state transition diagram in Figure 13 shows that there are no transient sets in the model, but only a single ergodic set with only one cyclic class. Hence, the properties of regular Markov chains can be exploited to analyze the behavior of the proposed model [16]. Let P be the regular transition probability matrix; then the steady state probability vector can be solved using the following equations:

(1) $$\pi P=\pi \;\text{and}\;\sum_{i=1}^{m}\pi_{i}=1$$

Here, m is the number of states and P is the fundamental matrix for the regular Markov chain. This is given by:

(2) $$Z=\left[Z_{\mathrm{ij}}\right]={\left(I-P+A\right)}^{-1}$$

where:

1. *A* is a limiting matrix determined by *P*, and the powers of *P^n^* approach the probability matrix *A*;
2. Each row of *A* consists of the same probability vector *π* = {*π*_1_, *π*_2_, … *π_n_*}, *i.e.*, *A* = ξ*π*, where ξ is the column vector with all entries equal to 1; and
3. *I* is the identity matrix.

The matrix *Z* can be used to study the behavior of the regular Markov chain, and, using this matrix, one can compute the mean number of times that the process is in a particular state.

Let *Y_j_*(*k*) be the number of times that a process is in state *S_j_* in the first *k* steps; then *M_i_*[*y*(*k*)], the mean number of times the process is in state *S_j_* starting from state *S_i_* is given by:

(3) $$M_{i}\left[y_{j}{}^{\left(k\right)}\right]\to \left(Z_{\mathrm{ij}}-\pi_{i}\right)+k\pi_{j}$$

The total number of boundary updates in *k* steps, starting from state *S_i_*, can be computed from the total number of times that the process is in the boundary states starting from state *S_j_*, the initial state. The average number of location updates (*U_bu_*) in the analytical model, is given by:

(4) $$U_{\mathrm{bu}}=M_{i}\left[y_{1}{}^{\left(k\right)}\right]+M_{i}\left[y_{2}{}^{\left(k\right)}\right]+M_{i}\left[y_{3}{}^{\left(k\right)}\right]+M_{i}\left[y_{4}{}^{\left(k\right)}\right]$$

so:

(5) $$U_{\mathrm{bu}}=\sum_{n=1}^{4}M_{i}\left[y_{n}{}^{\left(k\right)}\right]$$

where 1, 2, 3 and 4 are the boundary states in the model.

Generalizing:

(6) $$U_{\mathrm{bu}}=\sum_{n=1}^{\alpha }M_{i}\left[y_{n}{}^{\left(k\right)}\right]$$

We can use the above equation to determine the number of binding update messages. Because we need to send a binding update message whenever the sensor node moves between IP-WSNs, each time a node enters a boundary state, a binding update message is generated. Therefore, we need to determine the expected number of times that the process enters into a boundary state within k steps.

Thus, sensor nodes need to send *U_bu_* binding update messages, given that the sensor node experiences a total of k transitions between SMAGs. Therefore, the ratio of intra-IP-WSN mobility is denoted as *M*_int*ra*_*IP*−*WSN*_ and is expressed by:

(7) $$M_{\text{int}\mathrm{ra}\_\mathrm{IP}-\mathrm{WSN}}=\left(k-U_{\mathrm{bu}}\right)/k$$

The ratio of inter-IP-WSN mobility is denoted as *M*_int*er*_*IP*−*WSN*_ and is expressed by:

(8) $$M_{\text{int}\mathrm{er}\_\mathrm{IP}-\mathrm{WSN}}=U_{\mathrm{bu}}/k$$

### 4.3. Signaling Cost Analysis of IP-WSN

We have evaluated our proposed model based on signaling cost, mobility cost and energy consumption. In the subsequent section we discuss the signaling cost and energy consumption analysis. Mobility cost is evaluated based on signaling cost. To evaluate total signaling costs, we compare the results of the analytical model of IP-WSN with those of MIPv6 and PMIPv6. We already mentioned that the proposed global IP-WSN architecture is based on SPMIPv6 which is a sensor network-based PMIPv6 protocol.

**Figure 14.**
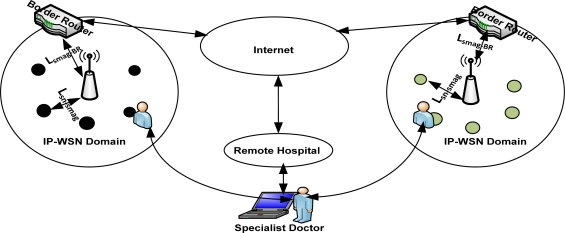
Analytical model for the performances analysis.

Figure 14 depicts the analytical model for the performance analysis of the proposed architecture. It consists of two different IP-WSN domains which are connected over the PMIPv6 based Internet. This figure is the analytical representation of Figure 1 which depicts the global IP-WSN architecture. We may consider the individual IP-WSN domain as the different floor of the patient care unit. Distance between SMAG and IP sensor node is denoted by D_sn-smag_ and the distance between SMAG and border router is denoted by D_smag-BR_. The corresponding node (CN) considered as the specialist doctor is communicating with the visiting doctor. There are some visiting doctors who see the patient on a continuous basis. In this analytical model, different distances are used for calculating signaling cost.

Signaling cost is defined as the total cost of signaling traffic overhead which in turn, is the total number of control messages exchanged between different nodes, so the unit of signaling cost is the number of control message exchanged among different nodes. There may be five types of mobility scenarios which has already been discussed in an earlier section. If patients move to a remote site then the signal will be transmitted through the Internet domain, otherwise signal transmission will be restricted within the IP-WSN domain.

From Equation (9) the total signaling cost (SigCost_mipv6_) of the proposed IP-WSN scheme based on MIPv6 is calculated by summing up the individual cost of intra-IP-WSN mobility (M_intra-ip-wsn_. C_sd_) and inter-IP-WSN mobility (M_inter-ip-wsn_.(C_sd_ + C_bu_)). Where, C_sd_ and C_bu_ are the sensor mobility cost and binding update cost respectively. Table 1 describes different symbols used in the equations.

(9a) $${\text{SigCost}}_{\text{mipv6}}=M_{\text{intra-ip-wsn}}.C_{\text{sd}}+M_{\text{inter-ip-wsn}}.\left(C_{\text{sd}}+C_{\text{bu}}\right)$$

where C_sd_ and C_bu_ are calculated in term of MIPv6 as follows:

(9b) $$C_{\text{sd}}=\alpha .\;\left({\text{RS}}_{\text{mipv6}}+{\text{RA}}_{\text{mipv6}}\right)\;D_{\text{sn-smag}}$$

(9c) $$C_{\text{bu}}=\;\alpha .\;\left({\text{BU}}_{\text{mipv6}}+{\text{BA}}_{\text{mipv6}}\right)\;D_{\text{sn-smag}}+\beta .\;\left({\text{BU}}_{\text{mipv6}}+{\text{BA}}_{\text{mipv6}}\right)D_{\text{smag-BR}}$$

From Equation (10) the total signaling cost (SigCost_pmipv6_) of the proposed IP-WSN scheme based on PMIPv6 is calculated by summing up the individual cost of intra-IP-WSN mobility (M_intra-ip-wsn_. C_sd_) and inter-IP-WSN mobility (M_inter-ip-wsn_.(C_sd_ + C_bu_)). Where, C_sd and_ C_bu_ are the sensor mobility cost and binding update cost respectively:

(10a) SigCostpmipv6=Mintra-ip-wsn. Csd+Minter-ip-wsn.(Csd+Cbu)

where Csd and Cbu are calculated in term of PMIPv6 as follows:

(10b) $$C_{\text{sd}}=\alpha .\left({\text{RS}}_{\text{pmipv6}}+{\text{RA}}_{\text{pmipv6}}\right)D_{\text{sn}}$$

(10c) $$C_{\text{bu}}=\beta .\left({\text{PBU}}_{\text{pmipv6}}+{\text{PBA}}_{\text{pmipv6}}\right)D_{\text{smag}}$$

From Equation (11) the total signaling cost (SigCost_spmipv6_) of the proposed IP-WSN scheme based on SPMIPv6 is calculated by summing up the individual cost of intra-IP-WSN mobility (M_intra-ip-wsn_. C_sd_) and inter-IP-WSN mobility (M_inter-ip-wsn_.(C_sd_ + C_bu_)). Where, C_sd and_ C_bu_ are the sensor mobility cost and binding update cost respectively:

(11a) $${\text{SigCost}}_{\text{spmipv6}}=M_{\text{intra-ip-wsn}}.C_{\text{sd}}+M_{\text{inter-ip-wsn}}.\left(C_{\text{sd}}+C_{\text{bu}}\right)$$

where C_sd_ and C_bu_ are calculated in term of SPMIPv6 as follows:

(11b) $$C_{\text{sd}}=\alpha .\left({\text{RS}}_{\text{spmipv6}}+{\text{RA}}_{\text{spmipv6}}\right)D_{\text{sn-smag}}$$

(11c) $$C_{\mathrm{bu}}=\beta .\left({\mathrm{PBU}}_{\mathrm{spmipv6}}+{\mathrm{PBA}}_{\mathrm{spmipv6}}\right)D_{\mathrm{smag-BR}}$$

### 4.4. Energy Consumption Analysis of IP-WSN

For energy consumption analysis, we consider an IP-WSN with densely deployed IP sensing devices. The network consists of two types of IP sensing device: fully functional IP sensing devices (IP-FFDs) and reduced functional IP sensing devices (IP-RFDs). IP-FFDs hold the complete 6LoWPAN protocol stack and perform the routing functions. IP-RFDs perform the sensing functions and forward the data to the IP-FFDs. We have followed the energy consumption model used in [7,19–23]. Table 2 represents the used parameter values in the energy consumption model. Here, *E^tx^* and *E^rx^* is the distance-independent amount of energy consumed by the transmitter and receiver electronics and the digital processing of each:

(12a) $$E=E^{\mathrm{tx}}+\beta *d^{\alpha }+E^{\mathrm{rx}}$$

Since same type of transmitting and receiving device is concerned in IP-WSN:

(12b) $$E^{\mathrm{tx}}=E^{\mathrm{rx}}=E_{\mathrm{dec}}$$

(12c) $$E=2*E_{\mathrm{dec}}+\beta *d^{\alpha }$$

For a particular fully functional device or a SMAG node *i*, control packet transmission cost can be calculated as follows:

(13) $$C_{i}^{\mathrm{ctrl}}\left(r\right)=\left[L_{\mathrm{ctrl}}*\beta *r^{\alpha }+\left({\mathrm{nrfd}}_{i}\left(r\right)+1\right)*L_{\mathrm{ctrl}}*L_{E}\right]\frac{1}{T}$$

Here *α* is path loss exponent (2 < *α* < 5) and *β* is a constant [J/bit m^2^], *r* is a transmission range. *nrfd_i_*(*r*) is the average number of reduced functional device nodes in the neighborhood of the fully functional node *i* within the range *r. L_ctrl_* is the length of control packets in bits, *L_E_* is the energy consumed by the transmitter device to transmit or receive a packet, and *T* is the time period between two consecutive topological changes of the IP-WSN.

Now for a particular path *p*, cost for information transmission from a source to the fully functional SMAG node *i* is calculated as follows:

(14) $$C_{i}^{\text{inf}}\left(p\right)=\left[\sum_{i=1,j=2}^{N}\left({\mathrm{nffd}}_{p}\left(d_{i}\right)+1\right)*L_{\mathrm{data}}*\beta d_{i,j}{}^{\alpha }+\left({\mathrm{nrfd}}_{p}\left(d_{i}\right)+1\right)*L_{\mathrm{data}}\right]*L_{E}$$

Here *nffd_p_*(*d_i_*) indicates the number of fully functional node for a path *p* and within the range *d. nrfd_p_*(*d_i_*) indicates the number of reduced functional device for path *p* and range *d*.

Total communication cost for transmitting data packet from a source to SMAG node *i* can be calculated from Equations (13) and (14):

(15) $$C_{i}^{\mathrm{total}}\left(p\right)=\sum_{i=1}^{N}\left[C_{i}^{\mathrm{ctrl}}\left(r\right)\right]+C_{i}^{\text{inf}}\left(p\right)$$

Now let us calculate the energy consumption in terms of MIPv6, PMIPv6 and the proposed global IP-WSN scheme based on SPMIPv6 with the help of Equations (9) to (15). Energy consumed by MIPv6 scheme can be calculated by the following mathematical derivation. Here $E_{i}^{\mathrm{mipv}6}$ indicates energy consumption by MIPv6 scheme:

(16) $$E_{i}^{\mathrm{mipv}6}=\mathrm{SigCos}t_{i}^{\mathrm{mipv}6}*C_{i}^{\mathrm{total}}\left(p\right)$$

Energy consumed by PMIPv6 scheme can be calculated by the following mathematical derivation. Here $E_{i}^{\mathrm{pmipv}6}$ indicates energy consumption by PMIPv6 scheme:

(17) $$E_{i}^{\mathrm{pmipv}6}=\mathrm{SigCos}t_{i}^{\mathrm{pmipv}6}*C_{i}^{\mathrm{total}}\left(p\right)$$

Finally energy consumed by the proposed IP-WSN based on SPMIPv6 scheme can be calculated in the same way. Here $E_{i}^{\mathrm{spmipv}6}$ indicates energy consumption by IP-WSN based on SPMIPv6 scheme:

(18) $$E_{i}^{\mathrm{spmipv}6}=\mathrm{SigCos}t_{i}^{\mathrm{spmipv}6}*C_{i}^{\mathrm{total}}\left(p\right)$$

## 5. Experimental Results

In this section, we present the result of experiments evaluating the performance of our proposed global IP-WSN scheme, and compare the performance of our proposed scheme to MIPv6 and PMIPv6. In the figures our proposed global IP-WSN scheme is represented as SPMIPv6. First, we evaluate the performance of our proposed approach by mathematical analysis. Then, we set signaling cost and mobility related cost for the number of IP based sensor nodes and number of hops traverse during the mobility phase in order to evaluate the consequences of our proposed scheme with PMIPv6 and MIPv6. Finally, we summarize the key characteristics of our proposed approach as compared to PMIPv6 & MIPv6 approach.

We have implemented the model and evaluate the parameter such as the signaling cost and mobility related cost as presented in this paper. The experiments were conducted on a computer equipped with an AMD Athlon™ 2.5 GHz CPU and 2 GB primary memory. We use the network simulator tool NS2 [24] on VMware based Linux Fedora 4. Now let us discuss the result with the figures.

**Figure 15.**
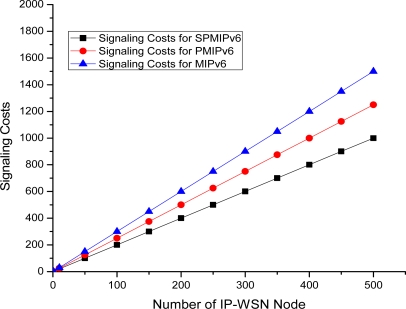
Number of IP-WSN nodes *vs.* Signaling Cost.

The Figure 15 depicts the signaling cost with respect to the number of IP-WSN nodes in term of the MIPv6, PMIPv6 and SPMIPv6. Signaling cost increases as the number of IP-WSN nodes increases. However, proposed IP-WSN scheme based on SPMIPv6 incurs much less signaling cost compared to MIPv6 and PMIPv6 as the number of IP-WSN nodes increases. The IP-WSN based SPMIPv6 scheme reduces signaling cost by 60% and 56% with respect to MIPv6 and PMIPv6 in terms of the number of IP-WSN nodes. The unit of signaling cost is the number of control messages exchanged for communication.

**Figure 16.**
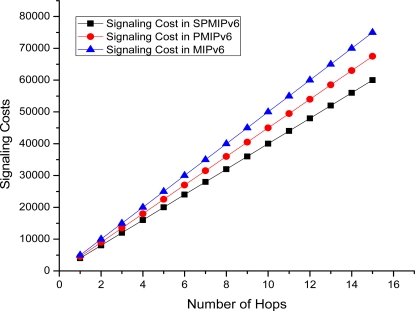
Number of Hops *vs.* Signaling Cost.

Figure 16 depicts the signaling cost with respect to the numbers of hops the data or signal traverses to reach the destination node. In this case we consider a maximum 15 hops. Signaling cost increases linearly as the number of hops increases. The proposed IP-WSN scheme based on SPMIPv6 shows better performance with respect to both PMIPv6 and MIPv6. In all cases signaling cost increases in a linear pattern. IP-WSN scheme based on SPMIPv6 reduces signaling cost by 56% and 53% with respect to MIPv6 and PMIPv6 in terms of the number of hops. The unit of signaling cost is the number of control messages exchanged for communication.

**Figure 17.**
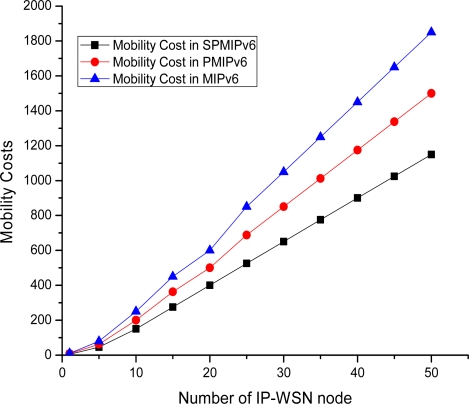
Number of IP-WSN nodes *vs.* Mobility Cost.

Figure 17 shows the mobility cost with respect to the number of mobile sensor nodes in MIPv6, PMIPv6 and IP-WSN based on SPMIPv6. Mobility cost increases as the number of mobile sensor nodes increases. IP-WSN based on SPMIPv6 enjoys less mobility cost with respect to MIPv6 and PMIPv6. Difference of mobility cost becomes more reasonable when number of mobile sensor nodes increases. The SPMIPv6 scheme reduces signaling cost by 62% and 57% with respect to MIPv6 and PMIPv6 in terms of the number of IP-WSN nodes. The unit of mobility cost is the number of control messages exchanged due to mobility.

**Figure 18.**
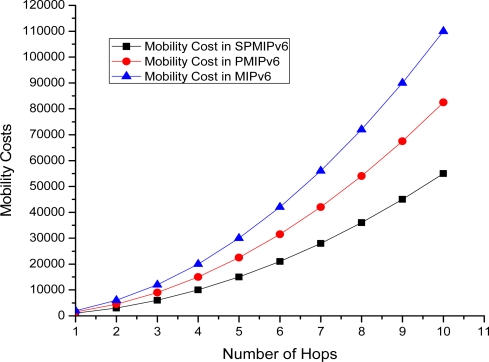
Number of Hops *vs.* Mobility Cost.

Figure 18 shows the mobility cost with respect to number of hops used by the mobile sensor nodes in MIPv6, PMIPv6 and IP-WSN based on SPMIPv6. Mobility cost increases exponentially as the number of hops increases. In this scenario also, our proposed IP-WSN scheme based on SPMIPv6 enjoys less mobility cost with respect to MIPv6 and PMIPv6. The proposed IP-WSN scheme based on SPMIPv6 scheme reduces 67% and 60% mobility cost with respect to MIPv6 and PMIPv6 in terms of the number of hops.

**Figure 19.**
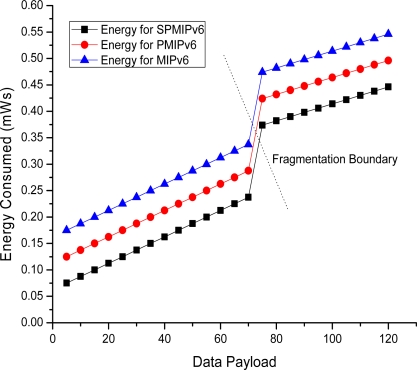
Data payload *vs.* Energy consumption.

Figure 19 shows the energy consumption with respect to payload. Energy consumption is linear in MIPv6, PMIPv6 and IP-WSN based on SPMIPv6. However, due to fragmentation overhead, energy consumption increased rapidly and then it represents the linear characteristics again. In this scenario, proposed IP-WSN scheme based on SPMIPv6 is energy efficient compared to other two approaches.

**Figure 20.**
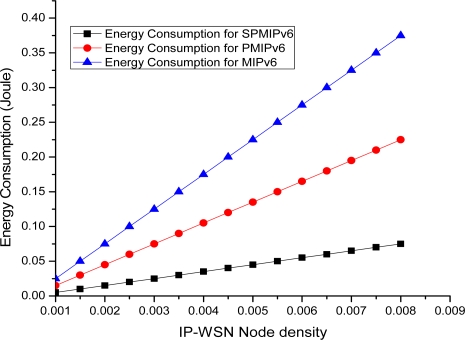
Node Density *vs.* Energy Consumptions.

**Figure 21.**
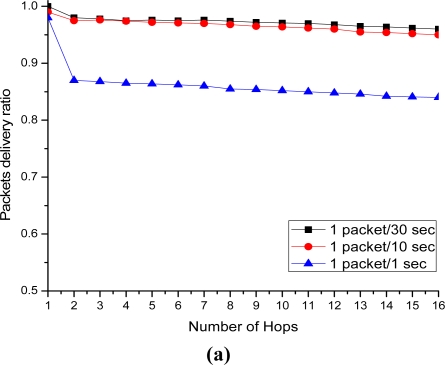

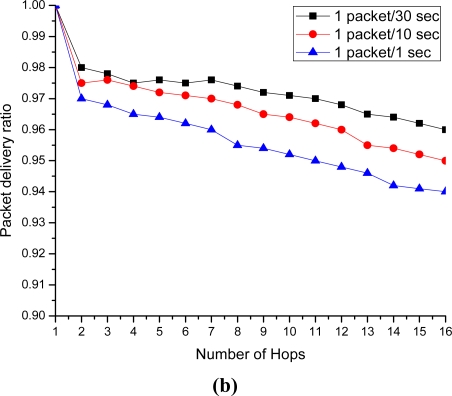
Delivery ratio *vs.* number of hops. (**a**) Multiple nodes to SMAG delivery; (**b**) End-to-end delivery.

Figure 20 depicts the energy consumption with respect to the IP-WSN node density in term of the MIPv6, PMIPv6 and the proposed IP-WSN based on SPMIPv6. Energy consumption increases as the IP-WSN node density increases. Our proposed scheme increases the performance linearly compared to MIPv6 and PMIPv6. Therefore, the energy consumption increases more rapidly as the density of IP-WSN node increases. Figure 21(a,b) represents packet delivery ratio versus number of hops. Packet delivery ratio is measured for 1 packet per 30 seconds, 1 packet per 10 seconds and 1 packet per one second with respect to the number of hops. Increasing number of hops between sources and destinations shows a small negative effect on packet delivery ratio for the IP-WSN scenario. The higher the traffic loads, however, the lower the packet delivery ratio. Figure 21(a) shows multiple nodes to SMAG node packet delivery ratio in IP-WSN. The delivery ratio is defined as the number of successfully delivered packets divided by the number of packets generated at sources. In this case the proposed routing and addressing scheme shows excellent delivery ratio except for 1 packet per second. In case of 1 packet per second traffic in which the end-to-end packet delivery suffers more contention and thus more MAC layer retransmission. Figure 21(b) considers a typical IP-WSN scenario where a single source node sends data periodically to a SMAG node over multiple hops. In this case also the proposed routing and addressing scheme shows excellent delivery ratio.

**Figure 22.**
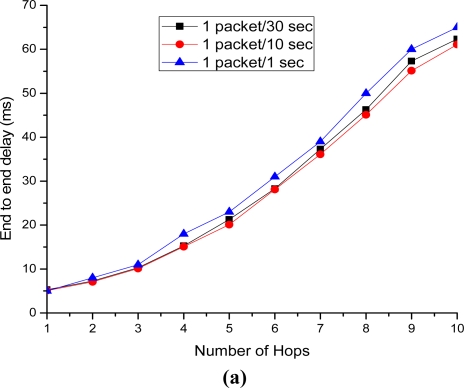

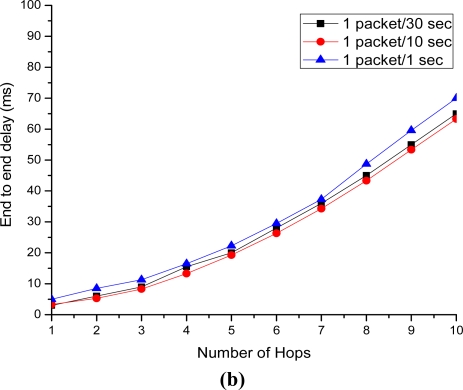
End to end delay *vs.* number of hops. (**a**) Multiple nodes to SMAG delivery dela; (**b**) End-to-end delay.

Figure 22(a,b) depict the end to end delay versus number of hops. Both figures show increasing amount of delay along with the increasing number of hops. The end-to-end delay is almost the same for both cases, except for the case of 1 packet per second traffic in which the end-to-end packet delivery delay suffers more contention and thus more MAC layer retransmission. Figure 22(a) considers a typical IP-WSN scenario where multiple nodes send data periodically to a SMAG node. Figure 22(b) is performed to show the end-to-end delay for end-to-end packet delivery scenario in IP-WSN. In both cases the proposed routing and addressing scheme minimize the end-to-end delay significantly.

## 6. Conclusions

In this paper we present a global IP-WSN architecture with special attention to addressing and routing. These are the most challenging issues that must be addressed with reference to energy efficiency. Since the proposed global IP-WSN architecture is based on a localized mobility management protocol, it meets the demand for energy efficiency in terms of reducing signaling costs, mobility costs, end to end delay and increasing packet delivery ratio. Here we also present the detailed software architecture, mobility scenarios, messages formats, addressing issues and evaluate its performance by analyzing signaling costs and mobility costs, packet delivery ratio and end to end delay. Our experiments show that with respect to the number of IP-WSN nodes, the proposed global IP-WSN scheme reduces the signaling cost by 60% and 56%, as well as the mobility cost by 62% and 57%, compared to MIPv6 and PMIPv6, respectively. The simulation results also show that in terms of the number of hops, proposed IP-WSN scheme based on SPMIPv6 decreases the signaling cost by 56% and 53% as well as mobility cost by 60% and 67% as compared to MIPv6 and PMIPv6 respectively. It also indicates that proposed scheme reduces the level of energy consumption. Moreover the proposed scheme also enhances the packet delivery ratio and end to end delay significantly. In this paper, we focus on 6LoWPAN based IP-WSN of the same vendor and protocol stack. In future we will focus on the federated IP based sensor networks consisting of multi-vendor and heterogeneous protocol stacks.

## Figures and Tables

**Table 1.** System Parameters.

**Symbol**	**Description**
BU	Binding Update Message
BA	Binding Acknowledgement Message
PBU	Proxy Binding Update Message
PBA	Proxy Binding Acknowledge Message
D_smag_BR_	Distance between SMAG and BR
D_sn_smag_	Distance between SN and SMAG
α	Unit transmission cost in a wireless link
β	Unit transmission cost in a wired link
RS	Router Solicitation Message
RA	Router Advertisement Message
C_sd_	Sensor Mobility Cost
C_bu_	Binding Update Cost

**Table 2.** Parameter values.

Parameter	Value
No of IP-WSN Node (N)	25∼120
Network Area (A)	120 × 120 M
Node density (ρ)	0.00173∼0.00833
Initial Energy	2 J
Transmit/Receive electronics (L_E_)	50 nJ bit^−1^ m^2^
Transmission Power	5.85 × 10^−5^ W
Number of SMAG	1–10
Transmission range (r)	25 m
Packet size	2 KB
